# 9. An unusual case of Takayasu arteritis presenting as leptomeningitis with obstructive hydrocephalus

**DOI:** 10.1093/rap/rky033.001

**Published:** 2018-09-20

**Authors:** Dhaval Tanna, Naval Mendiratta, Natasha Negalur

**Affiliations:** 1 Rheumatology, Medanta the Medcity Hospital, Haryana, INDIA; 2 Rheumatology, Fortis Memorial Research Institute, Haryana, INDIA

**Introduction:** Takayasu arteritis is a well-known, yet rare form of large vessel vasculitis with predominance in countries such as India and Japan. It is usually detected in young females aged 20-40, with the most common manifestation of claudication pain and fever of unknown origin. Neurological manifestations are common but rarely present as the first presenting symptom. The reported incidence of neurological manifestations including headache, dizziness, visual disturbance or loss of consciousness, stroke, and transient ischemic attack (TIA) varies from 57% up to 80%. Vasculitis can manifest primarily with striking leptomeningeal involvement as shown in various case reports of ANCA-associated vasculitis and primary CNS angitis. As Takayasu arteritis primarily affects proximal large vessels, leptomeningeal involvement seems theoretically unusual. Here, we report a case of nodular leptomeningitis as a presenting manifestation of Takayasu arteritis.

**Case description:** This is a case of a 23 year old female without any prior comorbidity who developed high-grade fever associated with headache and diffuse body ache in September 2017. Fever was not associated with sore throat, cough, burning micturition, altered bowel habits, night sweats or any other localising signs. She was started on azithromycin along with anti-pyretic, but continued to have daily spikes of fever. On the fifth day of illness, she developed altered sensorium. It was not preceded by excruciating headache or vomiting. It was not associated with seizure, arm/leg weakness or abnormal moments. She was taken to a higher centre where on examination she was found to have elevated blood pressure [210/150 mmHg]. She was started on intravenous anti-hypertensives and in view of altered sensorium; she underwent contrast enhanced computed tomography of the brain, which revealed hydrocephalus with dilated lateral and third ventricle and leptomeningeal enhancement. She was referred to a tertiary care centre for further evaluation and management. She was brought to the emergency department of our hospital on fifth day of illness. She was febrile and had elevated BP [200/110 mmHg] at time of admission despite being on intravenous anti-hypertensives. Her GCS was 6/15. Her general examination was unremarkable and respiratory and cardiovascular system examinations were normal. Neurological examination did not show any particular abnormality except bilateral extensor plantar response. She underwent MRI with contrast which showed nodular leptomeningeal enhancement in bilateral front parietal, occipital and right posterior temporal region. There were nodular enhancing foci in cerebellum, thalami, pons and midbrain. Fourth ventricle was compressed with resultant hydrocephalus and there was transtentorial herniation of left cerebellar hemisphere. Further history taking revealed history of pulmonary tuberculosis in a grandfather four months prior. Based on MRI findings and family history of tuberculosis exposure, probable diagnosis of tubercular meningitis was considered. She was intubated in view of deteriorating sensorium and was started on intravenous antibiotics (ceftriaxone 2 gms twice daily) and anti tubercular treatment (isoniazid, rifampicin, pyrizinamide, ethambutol and levofloxacin). In view of significant brain oedema and diagnosis of tubercular meningitis, intravenous steroids (dexamethasone 8 mg thrice daily) were added as well. She underwent external ventricular drainage procedure to relieve the hydrocephalus. Her drain showed clear cerebrospinal fluid which on cytological, biochemical and microbiological analysis did not reveal any significant abnormality (no evidence of mycobacterium tuberculosis). Her blood investigations revealed low Hb [8.6 gm/dl] and moderately elevated ESR and CRP [45 mm/hr and 36 mg/L]. Her Liver function, renal function, blood culture and urine cultures were sterile and 2 D echo was normal. At this point of time we were dealing with a case consisting of two cardinal issues: fever with altered sensorium for which possible explanation was tubercular leptomeningitis with obstructive hydrocephalus and accelerated hypertension in a young female. Rheumatology review was done in view of nodular enhancement in the brain with suspicion of neurosarcoidosis. After 48 hours of treatment, patient had improvement in sensorium and was extubated. This enabled us to take detailed history from patient herself. Patient said to be having on and off arthralgia and claudication pain in right upper limb for last one month. On re-examination, she was found to be having difference in pulse volume and blood pressure in both upper limbs. To substantiate the diagnosis of tubercular meningitis, repeat cerebrospinal fluid analysis was done which was normal. Her ANA, ENA, ANCA serology and RF were negative. Her serum ACE levels were normal. She was subjected to renal doppler as a part of evaluation of young hypertensive, which revealed left renal artery stenosis. She underwent contrast enhanced computed tomography of chest and abdomen with angiogram of abdominal aorta. It revealed wall thickening with luminal irregularity of infra renal aorta and right subclavian vessels. There was complete occlusion of proximal superior mesenteric artery, left renal artery and right external iliac artery along with small left kidney. Overall findings were suggestive of aortoarteritis. Meanwhile she kept on improving clinically due to intravenous steroids. Her external ventricular drain was removed and her blood pressure was controlled on a combination anti-hypertensive agents. She was switched to oral prednisolone 1 mg/kg. Her previous two CSF samples were taken from ventricular drains which were normal. To prove our diagnosis of tubercular meningitis, she was subjected to lumbar puncture and subsequent CSF analysis for the third time which was again normal. As her thrice repeated CSF samples were entirely normal, we became skeptical regarding our diagnosis of tubercular meningitis. But we were unable to explain nodular leptomeningitis and hydrocephalus with diagnosis of Takayasu arteritis alone. In literature review, we could not find any case describing leptomeningitis in patient with Takayasu arteritis. But we found case reports describing leptomeningitis in primary CNS angitis and ANCA-associated vasculitis. Case reports describing nodular enhancement in systemic lupus erythrematous, antiphospholipid syndrome and Behçet's disease were also found in the literature. In anticipation of reaching adiagnosis, we repeated MRI brain with MR angiogram of head & neck on day ten of admission. It revealed complete resolution of previously seen nodular lesions, significant decrease in oedema and mass effect, resolution of hydrocephalus and significantly narrowed bilateral middle cerebral artery and intracranial internal carotid artery. A mere course of ten days of anti tubercular treatment did not justify such a rapid resolution of nodular leptomeningitis. This enabled us to rule out coexistent tubercular meningitis. Anti tubercular therapy was discontinued. Based on adequate evidence of nodular leptomeningitis occurring in various forms vasculitis and intracranial vessel involvement well characterised in aortoarteritis patients presenting with neurological symptoms, a final diagnosis was Takayasu arteritis (type V) with nodular leptomeningitis with obstructive hydrocephalus was made. Patient was continued on tapering dose of oral steroid and steroid sparing immunosuppressant in the form of methotrexate was started on out patient visit. Patient has received regular follow-ups in the last eight months and is doing well.

**Discussion:** In Takayasu arteritis, involvement of carotid and vertebral arteries cause decreased cerebral blood flow and may present with symptoms ranging from light giddiness to stroke. While the extracranial neurovascular manifestations of Takayasu arteritis are well established, little is known regarding the intracranial manifestations. Intracranial vascular abnormalities in patients of Takayasu arteritis presenting with neurologic symptom are not rare and have been described in 15-20% patients presenting with neurological symptoms. Our patient had intracranial vessel involvement in form of bilateral middle cerebral and intracranial internal carotid artery narrowing. Leptomeningeal enhancement has been described in patients with primary CNS angitis and ANCA-associated vasculitis. Nodular brain lesions have been described in cerebral vasculitis secondary to SLE, anti phospholipid syndrome and Behcet disease. In our case, nodular leptomeningitis may represent diffuse inflammation and vasculitis of intracranial vessels in the cortex and deep white matter. Hydrocephalus in this case could be secondary to posterior fossa oedema caused hypertensive posterior reversible encephalopathy syndrome. In country with high prevalence of tuberculosis, tubercular meningitis remains the most common cause of leptomeningitis with hydrocephalus. But CSF abnormalities are present in almost all cases of tubercular leptomeningitis. Our case had normal CSF on three occasions and she had dramatic response to treatment in the form of resolution of nodular lesions on day ten of illness. Such dramatic resolution could only be due to the steroid therapy she was receiving rather than anti tubercular drugs. This supported Takayasu arteritis as primary diagnosis in our patient and presence of nodular leptomeningitis was thought to be secondary to intracranial vessel involvement.

**Key Learning Points:** Though involvement of proximal extra cranial vessels are more common in Takayasu arteritis, it may present with intracranial vascular involvement. Apart from ischemic manifestations, intra cranial vasculitis may manifest as leptomeningeal enhancement and nodular intracranial lesions. Accelerated hypertension may lead to posterior reversible encephalopathy syndrome which may cause posterior fossa oedema and hydrocephalus. In a young female, always examine blood pressure in all four limbs and look for renal and abdominal bruit.

**Disclosure: D. Tanna:** None. **N. Mendiratta:** None. **N. Negalur:** None.

